# Non-Orthogonal Multiple Access for Ubiquitous Wireless Sensor Networks

**DOI:** 10.3390/s18020516

**Published:** 2018-02-08

**Authors:** Asim Anwar, Boon-Chong Seet, Zhiguo Ding

**Affiliations:** 1 Department of Electrical and Electronic Engineering, Auckland University of Technology, Auckland 1010, New Zealand; asim.anwar@aut.ac.nz; 2 School of Computing and Communications, Lancaster University, Lancaster LA1 4YW, UK; z.ding@lancaster.ac.uk

**Keywords:** cross-technology interference, non-orthogonal multiple access, stochastic geometry, unlicensed spectrum, ubiquitous wireless sensor networks

## Abstract

Ubiquitous wireless sensor networks (UWSNs) have become a critical technology for enabling smart cities and other ubiquitous monitoring applications. Their deployment, however, can be seriously hampered by the spectrum available to the sheer number of sensors for communication. To support the communication needs of UWSNs without requiring more spectrum resources, the power-domain non-orthogonal multiple access (NOMA) technique originally proposed for 5th Generation (5G) cellular networks is investigated for UWSNs for the first time in this paper. However, unlike 5G networks that operate in the licensed spectrum, UWSNs mostly operate in unlicensed spectrum where sensors also experience cross-technology interferences from other devices sharing the same spectrum. In this paper, we model the interferences from various sources at the sensors using stochastic geometry framework. To evaluate the performance, we derive a theorem and present new closed form expression for the outage probability of the sensors in a downlink scenario under interference limited environment. In addition, diversity analysis for the ordered NOMA users is performed. Based on the derived outage probability, we evaluate the average link throughput and energy consumption efficiency of NOMA against conventional orthogonal multiple access (OMA) technique in UWSNs. Further, the required computational complexity for the NOMA users is presented.

## 1. Introduction

Non-orthogonal multiple access (NOMA) is emerging as a strong candidate for adoption as the multiple access technology to enhance system capacity for 5th Generation (5G) cellular systems [1,2]. In NOMA, the transmitted signals of multiple users are multiplexed in the power domain using same time, frequency or code resource, and demultiplexed by applying an interference cancellation technique at the receiver [3].

Although originally proposed for cellular systems, NOMA exhibits strengths that we consider as highly relevant to addressing the deployment challenges of ubiquitous wireless sensor networks (UWSNs), i.e., large-scale networks of wireless sensors densely deployed for ubiquitous monitoring of physical environments. Specifically, for a given spectrum bandwidth, NOMA can enable more simultaneous connections than existing approaches without the overheads of coding and spreading to facilitate the separation of users’ signals at the receiver [4]. This is particularly attractive for supporting massive connectivity without requiring more spectrum resources in UWSNs.

NOMA can be applicable to both uplink (sensors-to-sink) and downlink (sink-to-sensors) communication where powerful sink nodes can perform the equivalent role of base stations (BSs) for the tasks of user grouping and transmission power allocation. In UWSN, however, there is a greater motivation and challenge to apply NOMA in the downlink (DL).

Firstly, UWSNs with large geographic coverage typically use short-range multi-hop communication to conserve energy [5]. The size of the routing table at each node increases with the number of destinations. Unlike in uplink communication where the sink node is the main destination of all sensors’ outgoing traffic, the routing table size for DL communication can grow prohibitively with a massive number of sensors as destinations [6]. In this case, NOMA can offer a practical solution by enabling direct DL transmissions from sink node to multiple sensors simultaneously. DL transmissions are initiated when sink node queries a specific sensor or group of sensors for some information [7,8], or communicates information essential for their operations such as sleep-wake schedules [9]. Further, unlike 5G, most UWSNs operate in unlicensed spectrum where sensors also experience cross-technology (CT) interferences [10] from other devices sharing the same spectrum.

The main novelty and contributions of this paper are as follows. For the first time, NOMA is proposed as a spectrum efficient means of supporting massive connectivity in UWSNs, and its performance in a DL scenario where sink transmits to a group of sensors using NOMA under CT and other interferences is investigated using stochastic geometry [11]. The sensors, sinks and CT nodes can reside randomly and independently of each other in a two-dimensional (2D) plane. Hence, their spatial topologies can be modeled with three different homogeneous Poisson Point Processes (PPPs). We further derive the closed-form expression for the outage probability at probe receiver’s location and analyze its diversity order. We also present the average link throughput and energy consumption efficiency analysis to gain better understanding of applying NOMA to UWSN and benchmark the performance against conventional orthogonal multiple access (OMA). Numerical results are shown to validate the accuracy of the performed analysis as well as compare the outage, throughput and energy efficiency performances between the NOMA and OMA based UWSNs. Moreover, a computational complexity analysis is performed to evaluate the complexity required by SIC units of sensor receivers to decode NOMA message signals.

The rest of the paper is organized as follows. Section 2 reviews the related works. Section 3 describes the network model underlying our study. Section 4 derives the outage probability and diversity order at the sensor receiver under interference constraints. The network performances in terms of the average link throughput and energy consumption efficiency are then defined based on the derived outage probability in Section 5. Next, we present the numerical results in Section 6, and conclude the paper in Section 7.

## 2. Related Works 

Early studies have focused on the performance comparison between NOMA and orthogonal frequency division multiple access (OFDMA). Through system level simulations, the authors in [12] reported 30–40% gain in overall cell throughput for NOMA over OFDMA under frequency-selective wideband scheduling and power allocation.

In [13], the authors analyzed the performance of DL NOMA with randomly deployed users. They evaluated performance under two scenarios: i.e., when user’s quality of service (QoS) and user’s channel condition determine its rate. The results show that appropriate rate and power allocation would result in better outage and ergodic sum rate in NOMA than OMA.

The fairness achieved by DL NOMA is discussed in [14]. The authors proposed optimal power allocation coefficients based on the availability of perfect average channel state information. Results showed that NOMA with proposed power allocation maintains high fairness and achieves superior outage performance (user rate) compared to time division multiple access (TDMA) and NOMA with fixed power allocation.

The authors in [15] considered a NOMA employed in an underlay cognitive radio (CR) network. The secondary BS communicates with secondary users (SUs) using NOMA. The primary transmitters (PTs) spatial topology is modeled by a homogenous PPP in the primary network. The SU receptions are interfered by PTs transmissions. Stochastic geometry tools are then utilized to analyze interference and outage at the SU receiver. The results demonstrate that NOMA achieves lower outage probability compared to conventional OMA.

In [16], a multi-input multi-output (MIMO) NOMA framework based on the principle of signal alignment is proposed. The performance of MIMO NOMA system is evaluated under fixed and CR inspired power allocation strategies. Unlike conventional MIMO systems based on OFDM where a single sub-channel is allocated to one user, multiple users in MIMO NOMA system can share one sub-channel. The results show that the proposed framework can improve reception reliability of existing MIMO NOMA systems. The same authors further proposed a precoding design in [17] for MIMO NOMA in a DL scenario to suppress inter-cluster interference whereas intra-cluster interference is minimized using successive interference cancellation (SIC) technique. The authors in [18] analyzed the performance of MIMO NOMA and MIMO OMA systems when multiple users are grouped into clusters. Further, they proposed an admission control scheme to adaptively trade-off between sum rate and the number of users that can be admitted into a cluster. The results show that MIMO NOMA can outperform MIMO OMA in terms of both sum rate and user fairness.

NOMA with topological interference management (TIM) for single input single output (SISO) broadcast channel (BC) is proposed in [19]. The total *K* users are divided into *T* groups. The BS applies DL NOMA, which results in inter- and intra-group interferences. The authors applied the proposed TIM and SIC to minimize inter-group, and intra-group interference, respectively. Results showed that the proposed scheme achieves superior sum rate compared to traditional TDMA.

In [20], the authors analyzed a multi-cell uplink NOMA cellular network using stochastic geometry. The locations of BSs and cellular users are modeled by Poisson cluster process. Closed-form expressions for the Laplace transform of the interference at BS are derived by considering both intra- and inter-cluster interferences under various SIC scenarios. NOMA is shown to outperform OMA in terms of average rate coverage. The authors in [21] proposed a user scheduling scheme based on which a closed-form expression for power allocation is derived to maximize energy efficiency of downlink NOMA systems under imperfect channel state information. Results show that the proposed scheme can achieve higher energy efficiency than existing schemes and OMA. Similarly, in [22], the authors presented energy efficient user scheduling and power allocation schemes for NOMA with not only imperfect channel state information but also mutual cross-tier interference in heterogeneous networks. They studied the trade-offs between data rate and energy performances, and reported a high energy efficiency of NOMA in such networks. In [23], a joint subcarrier and power allocation method is proposed for NOMA based amplify-and-forward relaying that can secure communications in the presence of eavesdroppers through cooperative jamming. The proposed method shows enhanced NOMA energy efficiency and security over random resource allocation. More recently, the authors in [24] considered three cognitive NOMA architectures: underlay, overlay and CR-inspired NOMA architectures, and presented cooperative relaying strategies for mitigating inter- and intra-network interferences in order to improve their reception reliability.

To date, NOMA has been investigated only in the context of cellular networks. The use of NOMA for UWSN has not been proposed in literature, and the reported performance gains of NOMA over its counterparts cannot be straightforwardly claimed for UWSN. This is because, unlike in cellular network, the sink node in a UWSN has no control over all transmitters within its coverage, including sensors and sinks of other UWSNs under different administrative domains or CT nodes that share the same spectrum such as WiFi and Bluetooth devices. Hence, it is important to investigate the performance of a UWSN employing NOMA under interference-limited scenario.

## 3. Network Model

We consider a UWSN comprising of sensor and sink nodes, which are randomly distributed in an infinite 2D plane. Further, it is assumed that the CT nodes are also co-located with sensor and sink nodes. These CT nodes are not a part of UWSN but are operating in the same frequency band, and hence cause interference to the reception of probe receiver. The spatial topology of the sensor, sink and CT transmitter nodes is modeled by three homogenous PPPs, denoted by $\Xi_{SE}$, $\Xi_{SK}$ and $\Xi_{CT}$ with intensities $\lambda_{SE}$, $\lambda_{SK}$ and $\lambda_{CT}$, respectively.

**Figure 1:**
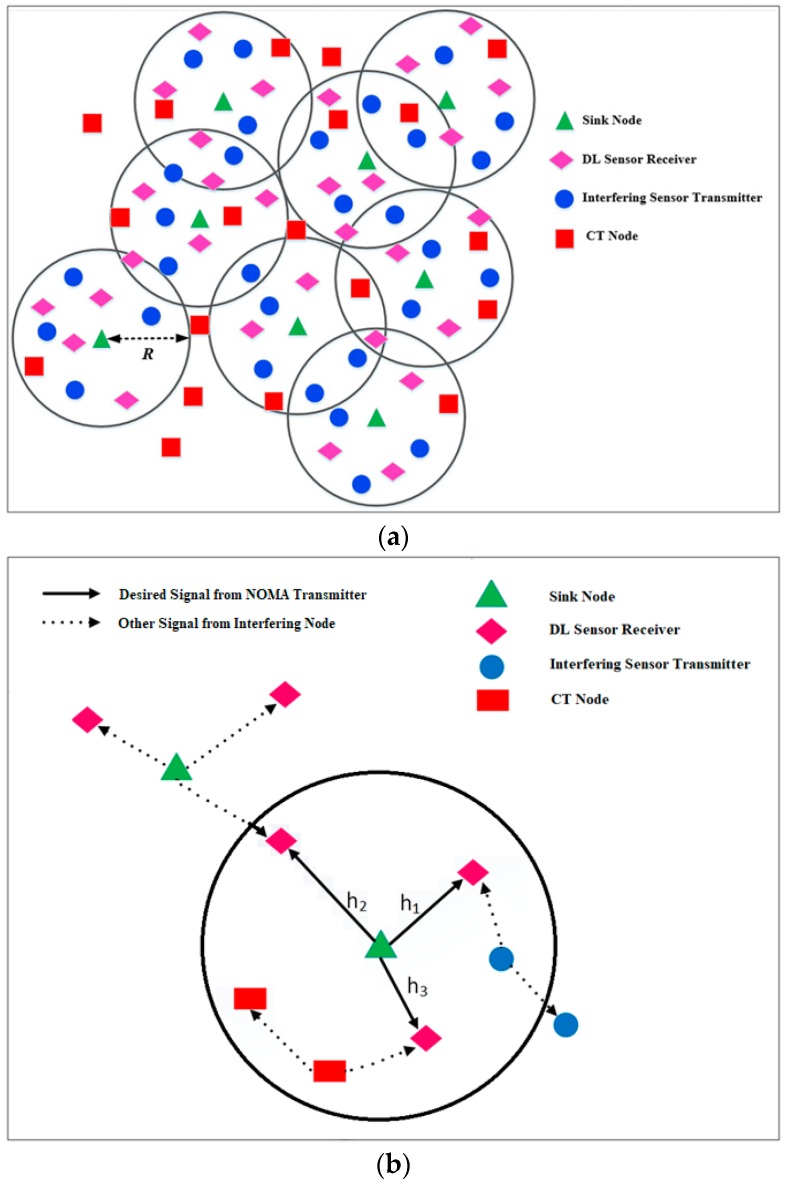
(**a**) A realization of $\Xi_{SE}$, $\Xi_{SK}$ and $\Xi_{CT}$ transmitter processes; and (**b**) an illustration of sink-to-sensors communication using NOMA under interference of other sink, sensor and CT nodes.

We focus on a DL transmission scenario where a sink node communicates with sensor nodes using NOMA. To avoid ambiguity, we refer to the sensor receivers as users. We also refer to the probe sink node as a test transmitter, which is considered to be located at the center of a disc A with radius R. The M NOMA users are considered to be uniformly distributed inside disc A, as shown in Figure 1. Further, by probe receiver, we always mean the *m*-th NOMA user.

All communication links in the network follow a composite Rayleigh fading and distance dependent path-loss channel model. The channel between the *m*-th user and test transmitter is given by $h_{m}={\hat{h}}_{m}{\left(1+d_{m}^{\alpha }\right)}^{-1/2}$, where ${\hat{h}}_{m}$ and $d_{m}$ represent the Rayleigh fading channel gain, and distance between the test transmitter and *m*-th user, respectively, and α is the path loss exponent. We use a bounded path loss model to avoid issue of singularity at small distances [11,13]. Without loss of generality, we assume NOMA users’ channel gains are ordered as ${\left|h_{1}\right|}^{2}\leq \ldots \leq {\left|h_{M}\right|}^{2}$. Consequently, the power allocation coefficients under NOMA are sorted as $\beta_{1}\geq \ldots \geq \beta_{M}$ with $\sum_{i=m+1}^{M}\beta_{i}=1$. The test transmitter sends a superimposed signal to all NOMA users and the received signal at the *m*-th user is given as:

(1) $$r_{m}=h_{m}\sum_{i=1}^{M}\sqrt{\beta_{i}P}s_{i}+n_{m},$$

where P is the transmission power of test transmitter, $s_{i}$ is the message signal of *i*-th sensor node, and $n_{m}$ is the additive white Gaussian noise (AWGN) with zero mean and variance $\sigma^{2}$.

For each NOMA user, there are two types of interference that interfere the reception of desired signal. First is the intra-user interference that is present due to the superposition of multiple users in NOMA, and the second is due to the transmission of undesired transmitters in the network. SIC is employed at each user receiver to mitigate the intra-user interference. The optimal decoding order of SIC is in the sequence of increasing channel gains. Therefore, *m*-th user decodes the message signals of all j users, j<m, before decoding its own message, and treats the message signals of the users j>m as noise.

For the second type, we consider that the interference links among unwanted transmitters and probe NOMA receiver are dominated by path loss. Assume the probe receiver is located at origin of the coordinate system i.e., $d_{m}=(x_{0},0)$ with $x_{0}\neq 0$, then the total interference at probe user’s location can be written as, $I=\sum_{w\in \Xi_{SE}}{\left(1+d_{w}^{\alpha }\right)}^{-1}+\sum_{x\in \Xi_{SK}\backslash x_{0}}{\left(1+d_{x}^{\alpha }\right)}^{-1}+\sum_{y\in \Xi_{CT}}{\left(1+d_{y}^{\alpha }\right)}^{-1}$, where $d_{w}$, $d_{x}$ and $d_{y}$ represent the distances between undesired transmitting sensors, sinks, CT nodes and the probe receiver, respectively. Further, sensors and sinks operate on very low power levels, thus the NOMA users may experience excessive interference from a nearby transmitter(s) leading to a situation of complete outage. To avoid this, we consider a guard zone of radius $d_{0}$ around each NOMA user, within which no unwanted transmitter is allowed to transmit [25].

A list of commonly used variables is summarized in Table 1.

Let $\Lambda_{m\to j}$ represents the signal-to-interference-and-noise ratio (SINR) at the *m*-th NOMA user to decode the message signal of *j*-th user, j<m, then $\Lambda_{m\to j}$ can be computed as follows:

(2) $$\Lambda_{m\to j}=\frac{{\left|h_{m}\right|}^{2}\beta_{j}\chi }{{\left|h_{m}\right|}^{2}\chi \sum_{i=j+1}^{M}\beta_{i}+\kappa I+1},$$

where $\chi ≜P/\sigma^{2}$ is the average system signal-to-noise ratio (SNR), $\kappa ≜P_{I}/\sigma^{2}$ is the average interference level, and $P_{I}$ is the common maximum transmission power available to sink, sensor and CT nodes.

If all the j<m users are decoded and removed successfully by the *m*-th user from its observation signal $r_{m}$, then SINR required to decode its own message is given by:

(3) $$\Lambda_{m\to m}=\frac{{\left|h_{m}\right|}^{2}\beta_{m}\chi }{{\left|h_{m}\right|}^{2}\chi \sum_{i=m+1}^{M}\beta_{i}+\kappa I+1}.$$

## 4. Outage and Diversity Analysis

### 4.1. Outage Probability Analysis

In this section, we present an exact analysis of the outage probability for *m*-th user under the considered network setting. Let $\varpi_{j}$ and $R_{j}$ represent the target SINR, and the rate for user j, respectively, where 1≤j≤M and $\varpi_{j}=2^{R_{j}}-1$. To simplify notation, we define $\Delta_{m,j}≜\left\{\Lambda_{m\to j}<\varpi_{j}\right\}$ as the outage event at *m*-th user when it fails to decode the message of *j*-th user, 1≤j≤m. Consequently, the outage probability for *m*-th user, denoted by $P_{\mathrm{out}}^{m}$, can be written as follows:

(4) $$P_{\mathrm{out}}^{m}=1-P_{r}(\Delta_{m,1}^{c}\cap \ldots \cap \Delta_{m,m}^{c}),$$

where $\Delta_{m,j}^{c}$ is the complement event of $\Delta_{m,j}$. To proceed further, rewrite $\Delta_{m,j}^{c}$ as:

(5) $$\begin{array}{cc} \Delta_{m,j}^{c} & =\left\{\Lambda_{m\to j}>\varpi_{j}\right\}=\left\{\frac{{\left|h_{m}\right|}^{2}\beta_{j}\chi }{{\left|h_{m}\right|}^{2}\chi \sum_{i=j+1}^{M}\beta_{i}+\kappa I+1}>\varpi_{j}\right\}\\ & =\left\{{\left|h_{m}\right|}^{2}\chi (\beta_{j}-\varpi_{j}\sum_{i=j+1}^{M}\beta_{i})>\varpi_{j}(\kappa I+1)\right\}\\ & \overset{\left(a\right)}{=}\left\{{\left|h_{m}\right|}^{2}>\tau_{j}(\kappa I+1)\right\}, \end{array}$$

where $\tau_{j}=\varpi_{j}{\left[\chi (\beta_{j}-\varpi_{j}\sum_{i=j+1}^{M}\beta_{i})\right]}^{-1}$. Step (a) provides the following essential condition to keep NOMA operational:

(6) $$C1:\beta_{j}-\varpi_{j}\sum_{i=j+1}^{M}\beta_{i}>0.$$

when condition C1 is violated, the *m*-th user will always suffer outage, irrespective of the channel SNR. Further, by defining $\tau_{m}^{*}=\max\{\tau_{1},\ldots ,\tau_{m}\}$, $P_{\mathrm{out}}^{m}$ can be written as:

(7) $$P_{\mathrm{out}}^{m}=1-\mathrm{Pr}\left({\left|h_{m}\right|}^{2}>\tau_{m}^{*}(\kappa I+1)\right)$$.

To proceed forward, we notice that ${\left|h_{m}\right|}^{2}$ are the ordered channel gains. Let $F_{X}(\cdot )$ and $f_{X}(\cdot )$ denote the cumulative distribution function (CDF), and the probability density function (PDF), of X respectively. Then, the ordered and unordered channel ${\left|\tilde{h}\right|}^{2}$ have following relationships [26]:

(8a) $$F_{{\left|h_{m}\right|}^{2}}(t)=\mu_{m}\sum_{q=0}^{M-m}\left(\begin{array}{c} M-m\\ q \end{array}\right)\frac{{\left(-1\right)}^{q}}{m+q}{\left[F_{|\tilde{h}{|}^{2}}(t)\right]}^{m+q}.$$

(8b) $$f_{{\left|h_{m}\right|}^{2}}(t)=\mu_{m}\sum_{q=0}^{M-m}\left(\begin{array}{c} M-m\\ q \end{array}\right){\left(-1\right)}^{q}{\left[F_{|\tilde{h}{|}^{2}}(t)\right]}^{m+q-1}f_{|\tilde{h}{|}^{2}}(t),$$

where $\mu_{m}=M!{\left[(M-m)!(m-1)!\right]}^{-1}$. To this end, the following theorem presents the exact expression for $P_{\mathrm{out}}^{m}$.

**Theorem 1.**  *The outage probability of the m-th NOMA user can be derived as:*

(9) Poutm=1Θ∑s=1SΨseυgs{∑k=02KRe[e−λπ[(e−ckd0−α−1)d02+ck∑t=1Tηte−utckd0−α]+iπgsΘ]×[μm∑q=0M−m(M−mq)(−1)q∑n=1Nψn(Φ(δ,1+δ;−an(κgs+1))+εΦ(1+δ,2+δ;−an(κgs+1)))×(1−e−τm*(κgs+1)tnΦ(δ,1+δ;−an(κgs+1)))m+q−1]},

*where* $\Psi_{s}=w_{s}e^{g_{s}}$*,* $w_{s}=\frac{\Gamma \left(S+1\right)g_{s}}{S!{\left(S+1\right)}^{2}{\left[L_{S+1}(g_{s})\right]}^{2}}$*,* $L_{S}(\cdot )$ *is the Laguerre polynomial of degree* S*,* $g_{s}$ *are the roots of* $L_{S}(\cdot ),$ $c_{k}=\upsilon_{0}+\frac{i\pi k}{\Theta }$*,* $\upsilon_{0}=\rho -\log(\xi )/\Theta$*,* ρ *is a real number,* ξ *is the desired relative accuracy,* Θ *is a scaling parameter,* $i=\sqrt{-1}$*,* K *is the number of terms used to invert the Laplace transform,* $\lambda =\lambda_{SE}+\lambda_{SK}+\lambda_{CT},$ $\eta_{t}=\frac{1}{2}d_{0}^{2-\alpha }\omega_{t}\sqrt{1-\phi_{t}^{2}}u_{t}^{-\delta }$*,* $\omega_{t}=\pi /T$*,* $u_{t}=(1+\phi_{t})/2,$ $\phi_{t}=\cos\left((2t-1)\pi /2T\right)$*,* $\psi_{n}=\omega_{n}\sqrt{1-\theta_{n}^{2}}\tau_{m}^{*}(\kappa z+1)e^{-\tau_{m}^{*}(\kappa z+1)t_{n}},$ $\theta_{n}=\cos\left((2n-1)\pi /2N\right)$*,* $\omega_{t}=\pi /N$*,* $t_{n}=(1+\theta_{n})/2,$ $a_{n}=\tau_{m}^{*}t_{n}R^{\alpha }$*,* δ=2/α*,* $\varepsilon =\delta {\left(1+\delta \right)}^{-1}R^{\alpha }$*, T and N are complexity-accuracy tradeoff parameters, and* Φ(⋅,⋅;⋅) *is a confluent hyper-geometric function.*

**Proof 1.**  See Appendix A. ☐

### 4.2. Diversity Analysis

In this subsection, we present the diversity analysis for the ordered NOMA users in high SNR regime. The diversity order of the *m*-th user outage probability is defined as:

(10) $$D=-\lim_{\chi \to \infty }\frac{\log P_{\mathrm{out}}^{m}}{\log\chi }.$$

To obtain *D*, we first notice from Equation (7) that the asymptotic outage probability in high SNR regime, denoted as $P_{m}^{\infty }$ can be expressed as:

(11) $$P_{m}^{\infty }=\mathrm{Pr}\left({\left|h_{m}\right|}^{2}<t^{*}\right),$$

where $t^{*}={\tilde{\tau }}_{m}\kappa I\chi^{-1}$, ${\tilde{\tau }}_{m}=\max\{{\bar{\tau }}_{1},\ldots ,{\bar{\tau }}_{m}\}$ and ${\bar{\tau }}_{m}=\varpi_{m}{\left[(\beta_{m}-\varpi_{m}\sum_{i=m+1}^{M}\beta_{i})\right]}^{-1}$. Next, when χ→∞ and $t^{*}\to 0$, similar to Equation (8a), the CDF of the ordered channel is expressed as:

(12) $$F_{{\left|h_{m}\right|}^{2}}^{\infty }(t^{*})=\mu_{m}\sum_{q=0}^{M-m}\left(\begin{array}{c} M-m\\ q \end{array}\right)\frac{{\left(-1\right)}^{q}}{m+q}{\left[F_{|\tilde{h}{|}^{2}}^{\infty }(t^{*})\right]}^{m+q}$$

We notice in the CDF of the unordered channel in Appendix A that $\Phi (\delta ,1+\delta ;-t^{*}R^{\alpha })\to 1$ and $e^{-t^{*}}\approx 1-t^{*}$. Hence, $F_{|\tilde{h}{|}^{2}}^{\infty }(t^{*})$ in Equation (12) can be approximated as:

(13) $$F_{|\tilde{h}{|}^{2}}^{\infty }(t^{*})\approx t^{*}$$

Substituting Equation (13) into Equation (12), $F_{{\left|h_{m}\right|}^{2}}^{\infty }(t^{*})$ can be expressed as:

(14) $$F_{{\left|h_{m}\right|}^{2}}^{\infty }(t^{*})\approx \vartheta {\left({\tilde{\tau }}_{m}\kappa I\chi^{-1}\right)}^{m}+o\left[{\left({\tilde{\tau }}_{m}\kappa I\chi^{-1}\right)}^{m}\right],$$

where $\vartheta =\mu_{m}/m$. Based on Equation (14), $P_{m}^{\infty }$ in (11) is given as:

(15) $$P_{m}^{\infty }\approx \frac{1}{\chi^{m}}\underset{\Psi }{\underset{︸}{\int_{0}^{\infty }\vartheta {\left({\tilde{\tau }}_{m}\kappa z\right)}^{m}f_{I}(z)dz}}$$

It can be observed that Ψ is a constant. Hence, Equation (15) can be expressed as follows:

(16) $$P_{m}^{\infty }\approx A\chi^{-m}+o(\chi^{-m}).$$

Finally, substituting Equation (16) into Equation (10), the diversity order experienced by the *m*-th user is found to be *m*. It can be observed from Equation (15) that interference in integral Ψ is independent of χ and hence the factor $\chi^{-m}$ dominates Ψ in Equation (15) under high SNR. This indicates that the interference-limited NOMA based UWSN becomes equivalent to an interference-free network. This result on diversity order can be interpreted as follows. First, the *m*-th user avails exactly *m* chances to decode its own message. Second, the *m*-th user has (*m*-1) interferences from the higher order users that need to be cancelled out by applying SIC. Hence, it obtains a diversity of *m*.

## 5. Throughput and Energy Consumption Efficiency

### 5.1. Link Throughput Efficiency

The link throughput efficiency between the *m*-th user and the test transmitter, denoted by $TP_{m}$ and measured in [bits/s/Hz] is defined as [27]:

(17) $$TP_{m}=(1-P_{\mathrm{out}}^{m})\log_{2}(1+\varpi_{m}).$$

Correspondingly, the average link throughput efficiency of the network can be found as:

(18) $$TP_{avg}=\left(\sum_{m=1}^{M}TP_{m}\right)/M$$

### 5.2. Energy Consumption Efficiency

NOMA is used by the sink node to communicate with the sensors. Since NOMA is typically applied on top of an underlying access technology to better reuse the transmission resources, e.g., time slots, frequency channels, or spreading codes, an additional SIC unit is required at the sensors to decode the desired message. It is thus important to analyze the overall energy consumption efficiency ς of the NOMA based UWSN. The total energy consumption along the signal path (communication and circuits) can be decomposed into three main components [28]: the power consumed by power amplifiers $P_{a}$, the power consumed by SIC unit to process information $P_{s}$, and the power consumed by all other circuit units (filters, mixers, frequency synthesizer, etc.) $P_{c}$. Hence, the overall ς in Joules/bit can be expressed as:

(19) $$\varsigma =\left(P_{a}+MP_{c}+P_{s}\right)/R_{b}$$

where $R_{b}=B\cdot TP_{avg}$ is the bit rate, B is the channel bandwidth in Hz, $P_{a}=\nu P$, $\nu =\nu_{2}/\nu_{1}$, $\nu_{1}$ is the drain efficiency of the power amplifier, and $\nu_{2}$ is the peak-to-average ratio.

The $P_{c}$ is considered as a constant, and $P_{s}$ can be regarded as the average power consumed by SIC units of all scheduled sensor receivers. To find $P_{s}$, we need to compute the power consumed by *m*-th sensor, $P_{m}$, to process $N_{b}=\left|B\cdot TP_{m}\right|$ bits. Our approach is to express $P_{m}$ in terms of the required computational complexity $C_{m}$.

To proceed forward, we first require a power consumption model for the sensor device. Considering the sensor as CMOS device, the power consumed during computation in a static CMOS device is given as [29]:

(20) $$P_{m}=C_{eff}V_{m}^{2}\hbar_{m},$$

where $C_{eff}$, $V_{m}$ and $\hbar_{m}$ are the effective switching capacitance, supply voltage, and clock frequency of *m*-th sensor, respectively. Further, $V_{m}$ and $\hbar_{m}$ are directly related as:

(21) $$\hbar_{m}=\partial V_{m},$$

where ∂>0 is a design parameter that takes a value of O(B) [30]. Dynamic voltage scaling (DVS) is a standard technique for conserving power in CMOS devices through dynamically adjusts the clock frequency by scaling the voltage according to processing load [31]. For a given *N_b_*, $V_{m}$ can be scaled to match $\hbar_{m}$ with $C_{m}$/s. Based on Equations (20) and (21), $P_{m}$ can be expressed as:

(22) $$P_{m}=C_{eff}{\left[\frac{C_{m}}{\partial }\right]}^{3}$$

and *C_m_* is given in terms of number of floating point operations (FLOPs) per bit decision [32] as:

(23) $$\begin{array}{cc} C_{m} & =N_{b}[2N_{s}M+5M+8\sum_{m=1}^{M-1}m+(M-1)(5+2N_{s})]\\ & +2MN_{s}(M-1)+2MN_{b}+M+M\log_{2}(M) \end{array}$$

where $N_{s}$ is the number of samples to represent one bit. Finally, based on Equations (22) and (23), substituting $P_{s}=M^{-1}\sum_{m=1}^{M}P_{m}$ into Equation (19) obtains the overall ς.

## 6. Numerical Results

This section presents results to verify the accuracy of outage probability, link throughput and energy consumption analysis for a UWSN which uses NOMA for downlink (sink-to-source) transmission. We calculate the coefficients $\beta_{m}$ according to the power allocation scheme proposed for NOMA in [13], with $\beta_{m}=(M-m+1)/\mu ,$ where μ is selected such that $\sum_{m=1}^{M}\beta_{m}=1$. In all the simulations, we use parameter values in Table 2 unless otherwise stated.

**Figure 2:**
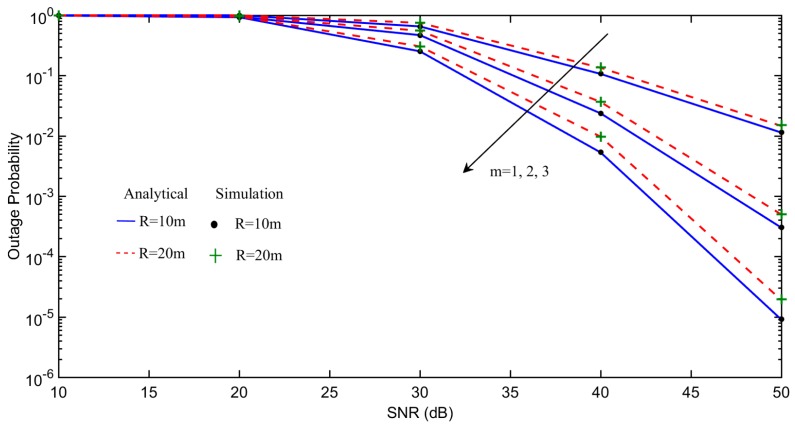
Impact of R on outage probability of each *m*-th user.

Figure 2 shows the outage probability of each *m*-th user under the impact of different transmission radius R of the sink node. The solid and dashed curves are analytical results obtained by plotting (9), while simulation results are shown in “●” and “+” to validate our derivations. It can be observed that increasing R results in higher outage probability due to increased path loss. Further, the ordered NOMA users have different outage performance, as each has a different channel condition.

**Figure 3:**
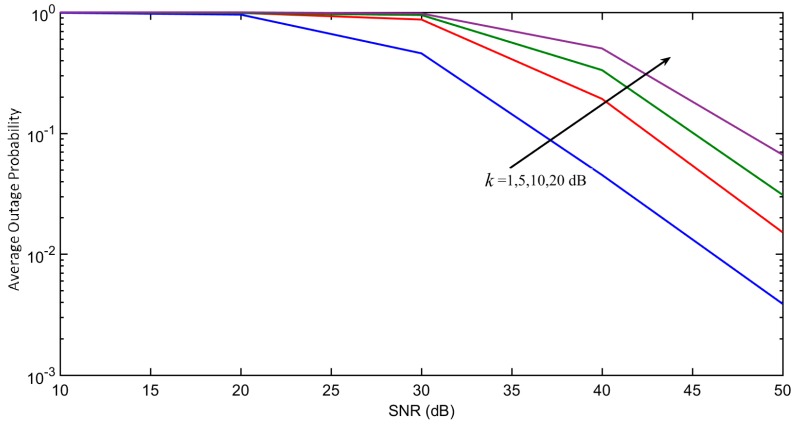
Impact of κ on average outage probability. The bottom first line (blue) is for k = 1 dB, second line (red) is for k = 5 dB, third line (green) is for k = 10 dB, and fourth line (purple) is for k = 20 dB, ordered by the direction of the arrow.

**Figure 4:**
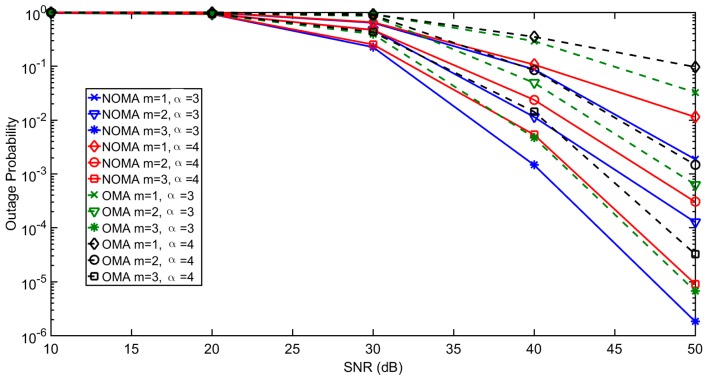
Impact of α on outage probability of each *m*-th user.

**Figure 5:**
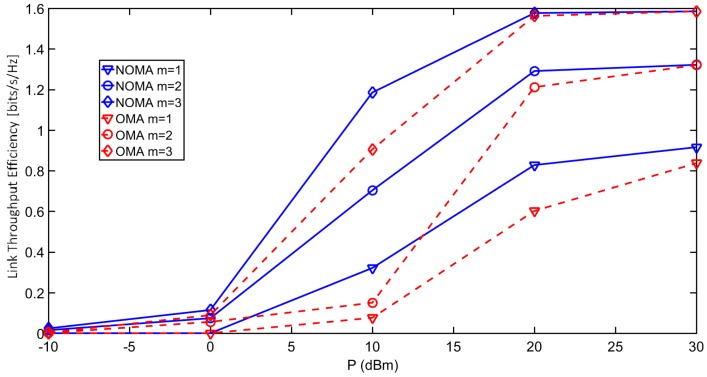
Link throughput efficiency comparison between NOMA and OMA.

Figure 3 demonstrates the average outage probability under different interference levels κ, defined immediately after Equation (2). Reducing κ expectedly lowers the outage probability. Figure 4 further shows the outage probability of each *m*-th user under the impact of different path loss α. The results show that NOMA achieves lower outage probability than OMA for different values of α. The link throughput efficiency of each *m*-th user with increasing transmit power of test transmitter (sink node) are shown in Figure 5. NOMA achieves better throughput efficiency than OMA, as all the ordered NOMA users have lower outage probability than their OMA counterparts.

**Figure 6:**
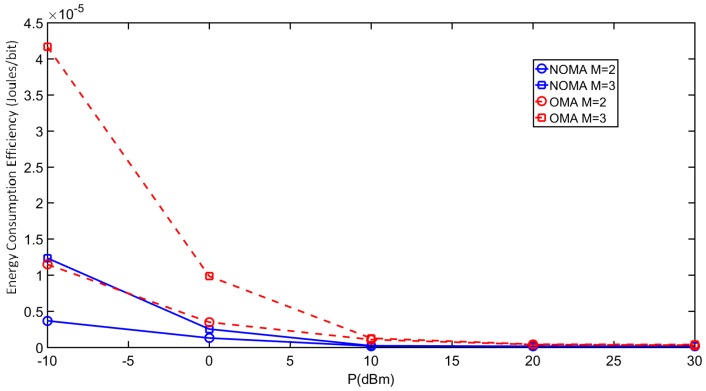
Energy consumption efficiency comparison between NOMA and OMA.

Figure 6 shows the energy consumption efficiency ς comparison between NOMA and OMA as a function of transmit power *P*. The results are obtained by considering two and three users. The power allocation coefficients and SINR thresholds for M = 3 are given in Table 2. For *M* = 2, they are chosen as $\beta_{m}={\left\{0.8,0.2\right\}}_{m=1,2}$, and $\varpi_{m}={\left\{0.9,1.5\right\}}_{m=1,2}$ respectively. Further, $C_{eff}=2\times 10^{-15}$ Farads for 70 nm CMOS technology [33], channel bandwidth B=2 MHz, $N_{s}=4$ samples to represent one bit, and circuit power consumption $P_{c}$ = 20 dBm. Overall, NOMA achieves better ς than OMA due to a lower outage probability which results in larger $R_{b}$ in Equation (19) and consequently higher ς. However, the ς of both schemes are comparable beyond a transmit power of 15 dBm, which indicates the importance of optimizing the power and rate allocation in NOMA based UWSNs.

Another observation is that, for both NOMA and OMA, the ς with *M* = 2 are better that with *M* = 3 at low transmit powers. This can be explained as follows. First, compared to *M* = 3, the $MP_{c}$ term in Equation (19) is lower for *M* = 2 resulting in better ς. Second, at low transmit powers, NOMA with *M* = 2 achieves higher $TP_{avg}$ than that with *M* = 3 because of less intra-user interference. Similarly, OMA with *M* = 2 has better *TP_avg_* than that with *M* = 3 because a higher fraction of B is available to each OMA user.

**Figure 7:**
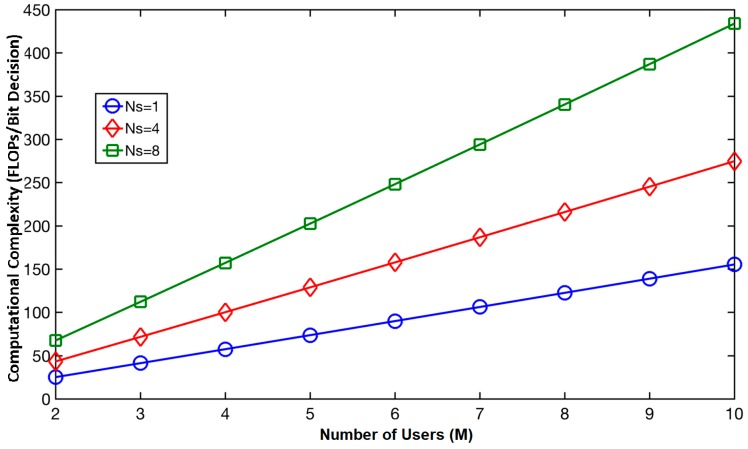
Computational complexity of NOMA receivers.

Since sensors have limited processing capacity, it is necessary to also analyze the complexity requirements of NOMA for these sensors. For downlink (sink-to-sensors) communication, the required complexity for the scheduled sensor receivers will be the complexity of their SIC units to process the received NOMA message. Figure 7 shows the computation complexity in terms of the number of floating-point operations (FLOPs) per bit decision, i.e., the number of FLOPs needed to decode one bit, obtained using Equation (23), under different *M* ordered users and *N_s_* samples used to represent one bit. The results expectedly show the receiver’s complexity increases with *M* and *N_s_*. 

To evaluate whether the current sensor platforms can implement the SIC unit for NOMA in UWSNs, we consider ARM Cortex-M3 and Cortex-M7 processors, which are widely used processors for current lower-, and higher-end sensor platforms, respectively. Based on their specified capacity in terms of million floating-point operations per second (MFLOPS) [34], we calculate the required computational time per bit decision for *M* = 3 and *M* = 9 users with *N_s_* = 4 samples, as shown in Table 3. Consider a maximum payload size of 127 bytes (or 1016 bits) widely used in low-powered UWSNs, the computational time per message with *M* = 3–9 users can range 115–411 ms, and 3.3–11.5 ms for Cortex-M3, and Cortex-M7 processor, respectively. 

## 7. Conclusions

In this paper, we propose and investigate the performance of NOMA for UWSNs. Different from cellular use-case, NOMA in UWSNs are further subject to interferences from cross-technology nodes operating in the same unlicensed spectrum as the sensors. Focusing on the downlink (sink-to-sensors) scenario, we derived a new closed-form expression for outage probability at the probe receiver’s location by utilizing stochastic geometry and order statistics. Numerical analysis shows that NOMA achieves lower outage probability, resulting in higher average throughput and better energy consumption efficiency than conventional OMA, suggesting that NOMA is very attractive for interference-limited UWSNs. Further, the computational time complexity for NOMA message decoding is within acceptable limits when using current and upcoming generations of processors for UWSNs. For future work, we plan to investigate power and rate allocation strategies under similar channel conditions to further enhance NOMA performance in UWSNs.

## Figures and Tables

**Table 1:** Commonly used variables.

Notation	Description
α	Path loss exponent
$\beta_{j}$	Power allocation coefficient of *j*-th user
$\varpi_{j}$	Target SINR threshold for *j*-th user
$P_{\mathrm{out}}^{m}$	Outage probability of the *m*-th user
Φ(⋅,⋅;⋅)	Confluent hyper-geometric function
$\Lambda_{m\to j}$	SINR at m-th user to decode j-th user message
$\Lambda_{m}$	SINR at m-th user to decode its own message
κ	Average interference level
χ	Average system SNR
$d_{m}$	Distance between test transmitter and *m*-th user
$d_{0}$	Guard zone radius around each sensor receiver
$h_{m}$	Channel between *m*-th NOMA user and test transmitter
M	Total number of NOMA users
$L_{S}(\cdot )$	Laguerre polynomial of degree S
N,T	Gaussian–Chebyshev parameters
P	Transmit power of test transmitter
$P_{c}$	Constant power consumption of circuits
R	Radius of disc A
$TP_{m}$	Average link throughput of user *m*
ς	Overall energy consumption efficiency

**Table 2:** Simulation parameters.

Parameter	Description	Value (s)
α	Path loss exponent	4
$\beta_{m}$	Power allocation coefficient of *m*-th user	${\left\{0.6,0.3,0.1\right\}}_{1,2,3}$
$\varpi_{m}$	SINR threshold for *m*-th user	${\left\{0.9,1.5,2\right\}}_{1,2,3}$
$\lambda_{SE}$	Intensity of $\Xi_{SE}$	$10^{-3}$
$\lambda_{SK}$	Intensity of $\Xi_{SK}$	$10^{-4}$
$\lambda_{CT}$	Intensity of $\Xi_{CT}$	$10^{-3}$
κ	Average interference level	1 dB
$\sigma^{2}$	Noise power	−90 dBm
χ	Average system SNR	10 to 50 dB
K	Fourier series terms	10
M	Total NOMA users	3
N	Gaussian–Chebyshev parameter	3
P	Test transmitter transmission power	−10 to 30 dBm
R	Transmission radius of test transmitter	10 m
S	Degree of Laguerre polynomial	2
T	Gaussian–Chebyshev parameter	5

**Table 3:** Computational time per bit decision by SIC unit.

Processor	Capacity (MFLOPS)	Time (μs) for *M* = 3	Time (μs) for *M* = 9
ARM Cortex-M3	0.618	113	405
ARM Cortex-M7	22.1	3.2	11.3

## Appendix A. Proof of Theorem 1

(A1) $$\begin{array}{c} P_{\mathrm{out}}^{m}=1-\mathrm{Pr}\left({\left|h_{m}\right|}^{2}>\tau_{m}^{*}(\kappa I+1)\right)\\ =\int_{0}^{\infty }\underset{Q1}{\underset{︸}{\int_{0}^{\tau_{m}^{*}(\kappa z+1)}f_{{\left|h_{m}\right|}^{2}}(x)dx}}f_{I}(z)dz, \end{array}$$

where $f_{I}(z)$ is the PDF of interference I. Since NOMA users are uniformly distributed inside disc A and fading is Rayleigh distributed, the CDF of the unordered channel is given as [35]:

(A2) $$\begin{array}{c} F_{{\left|\tilde{h}\right|}^{2}}(x)=\frac{2}{R^{2}}\int_{0}^{R}\left(1-e^{-(1+t^{\alpha })x}\right)tdt\\ F_{{\left|\tilde{h}\right|}^{2}}(x)\overset{\left(b\right)}{=}\frac{2}{\alpha R^{2}}\int_{0}^{R^{\alpha }}\left(1-e^{-(1+y)x}\right)y^{\delta -1}dy\\ \overset{\left(c\right)}{=}1-\delta e^{-x}B(1,\delta )\Phi (\delta ,1+\delta ;-xR^{\alpha })\\ =1-e^{-x}\Phi (\delta ,1+\delta ;-xR^{\alpha }), \end{array}$$

where (b) is obtained by a change of variable from $t^{\alpha }\to y$, (c) results from by applying ([36], Equation 3.383), B(⋅,⋅) is a beta function and δB(1,δ)=1. The PDF $f_{|\tilde{h}{|}^{2}}(x)$ is obtained by taking the derivative of (A2) as:

(A3) $$f_{|\tilde{h}{|}^{2}}(x)\overset{\left(d\right)}{=}e^{-x}\left[\Phi (\delta ,1+\delta ;-xR^{\alpha })+\varepsilon \Phi (1+\delta ,2+\delta ;-xR^{\alpha })\right]$$

where (d) is obtained by applying ([36], Equation 9.213) and $\varepsilon =\delta {\left(1+\delta \right)}^{-1}R^{\alpha }$. Next, using Equations (8b), (A2) and (A3), $Q_{1}$ in Equation (A1) can be written as:

(A4) $$\begin{array}{c} Q_{1}=\Omega_{m}\int_{0}^{\tau_{m}^{*}(\kappa z+1)}{\left[1-e^{-x}\Phi (\delta ,1+\delta ;-xR^{\alpha })\right]}^{m+q-1}\\ \times e^{-x}\left[\Phi (\delta ,1+\delta ;-xR^{\alpha })+\varepsilon \Phi (1+\delta ,2+\delta ;-xR^{\alpha })\right]dx, \end{array}$$

where $\Omega_{m}=\mu_{m}\sum_{q=0}^{M-m}\left(\begin{array}{c} M-m\\ q \end{array}\right){\left(-1\right)}^{q}$. The Gaussian–Chebyshev quadrature is applied to approximate $Q_{1}$ as:

(A5) $$\begin{array}{cc} Q_{1}(z) & =\Omega_{m}\{\sum_{n=1}^{N}\psi_{n}\left[\Phi (\delta ,1+\delta ;-a_{n}(\kappa z+1))+\varepsilon \Phi (1+\delta ,2+\delta ;-a_{n}(\kappa z+1))\right]\\ & \times {\left[1-e^{-\tau_{m}^{*}(\kappa z+1)t_{n}}\Phi (\delta ,1+\delta ;-a_{n}(\kappa z+1))\right]}^{m+q-1}\}. \end{array}$$

Next, the following lemma provides the PDF $f_{I}(z)$ of the interference to obtain $P_{\mathrm{out}}^{m}$.

**Lemma 1.** *Consider the interferers are distributed according to the homogenous PPP. Then the PDF of the interference at probe receiver can be given as:*

(A6) fI(z)=eυzΘ∑k=02KRe{e−λπ[(e−ckd0−α−1)d02+ck∑t=1Tηte−utckd0−α]+iπzΘ},

**Proof 2.** Please see Appendix B. ☐

Now, based on Equations (A5) and (A6), the outage probability for *m*-th user in Equation (A1) can be written as:

(A7) Poutm=∑k=02K∫0∞Re{e−λπ[(e−ckd0−α−1)d02+ck∑t=1Tηte−utckd0−α]+iπzΘ}eυzΘQ1(z)dz︸Q2.

As it is challenging to solve $Q_{2}$ in Equation (A7), we approximate it by Gauss–Laguerre quadrature as follows:

(A8) Q2=∑s=1SΨsRe{e−λπ[(e−ckd0−α−1)d02+ck∑t=1Tηte−utckd0−α]+iπgsΘ}eυgsΘQ1(gs),

Finally, substituting Equation (A8) into Equation (A7) and using $Q_{1}(z)$ in Equation (A5) with $z=g_{s}$, prove the result in Theorem 1.

## Appendix B. Proof of Lemma 1

To derive the PDF of interference I, we first notice that the Laplace transform of I is given as [25]:

(A9) $$L_{I}(s)=e^{-\lambda \pi \left[\left(e^{-sd_{0}^{-\alpha }}-1\right)d_{0}^{2}+s^{\delta }\gamma \left(1-\delta ,sd_{0}^{-\alpha }\right)\right]},$$

where $\gamma (a,x)=\int_{0}^{x}e^{-t}t^{a-1}dt$ is the lower incomplete gamma function. Next, we approximate $\gamma (1-\delta ,sd_{0}^{-\alpha })$ by applying the Gaussian–Chebyshev quadrature:

(A10) $$\gamma (1-\delta ,sd_{0}^{-\alpha })=s^{1-\delta }\sum_{t=1}^{T}\eta_{t}e^{-u_{t}sd_{0}^{-\alpha }}.$$

Substituting Equation (A8) into Equation (A7), $L_{I}\left(s\right)$ can be written as:

(A11) $$L_{I}(s)=e^{-\lambda \pi \left[\left(e^{-sd_{0}^{-\alpha }}-1\right)d_{0}^{2}+s\sum_{t=1}^{T}\eta_{t}e^{-u_{t}sd_{0}^{-\alpha }}\right]}.$$

Applying Fourier series [37] to invert the Laplace transform of the interference, the PDF $f_{I}(z)$ can be written as:

(A12) fI(z)=eυzΘ∑k=02KRe[LI(s)eiπzΘ].

Replacing s in Equation (A11) with $c_{k}$ and then substituting it into Equation (A12) prove Lemma 1.
